# Preventing Childhood Malaria in Africa by Protecting Adults from Mosquitoes with Insecticide-Treated Nets

**DOI:** 10.1371/journal.pmed.0040229

**Published:** 2007-07-03

**Authors:** Gerry F Killeen, Tom A Smith, Heather M Ferguson, Hassan Mshinda, Salim Abdulla, Christian Lengeler, Steven P Kachur

**Affiliations:** 1 Ifakara Health Research and Development Centre, Ifakara, Morogoro, United Republic of Tanzania; 2 Department of Biological and Biomedical Sciences, University of Durham, Durham, United Kingdom; 3 Department of Public Health and Epidemiology, Swiss Tropical Institute, Basel, Switzerland; 4 Division of Infection and Immunity, Glasgow University, Glasgow, United Kingdom; 5 Division of Environmental and Evolutionary Biology, Glasgow University, Glasgow, United Kingdom; 6 United States Public Health Service Commissioned Corps and Malaria Branch, Division of Parasitic Diseases, Centers for Disease Control and Prevention, Atlanta, Georgia, United States of America; Princeton University, United States of America

## Abstract

**Background:**

Malaria prevention in Africa merits particular attention as the world strives toward a better life for the poorest. Insecticide-treated nets (ITNs) represent a practical means to prevent malaria in Africa, so scaling up coverage to at least 80% of young children and pregnant women by 2010 is integral to the Millennium Development Goals (MDG). Targeting individual protection to vulnerable groups is an accepted priority, but community-level impacts of broader population coverage are largely ignored even though they may be just as important. We therefore estimated coverage thresholds for entire populations at which individual- and community-level protection are equivalent, representing rational targets for ITN coverage beyond vulnerable groups.

**Methods and Findings:**

Using field-parameterized malaria transmission models, we show that high (80% use) but exclusively targeted coverage of young children and pregnant women (representing <20% of the population) will deliver limited protection and equity for these vulnerable groups. In contrast, relatively modest coverage (35%–65% use, with this threshold depending on ecological scenario and net quality) of all adults and children, rather than just vulnerable groups, can achieve equitable community-wide benefits equivalent to or greater than personal protection.

**Conclusions:**

Coverage of entire populations will be required to accomplish large reductions of the malaria burden in Africa. While coverage of vulnerable groups should still be prioritized, the equitable and communal benefits of wide-scale ITN use by older children and adults should be explicitly promoted and evaluated by national malaria control programmes. ITN use by the majority of entire populations could protect all children in such communities, even those not actually covered by achieving existing personal protection targets of the MDG, Roll Back Malaria Partnership, or the US President's Malaria Initiative.

## Abstract 2

Based on malaria transmission models, Gerry Killeen and colleagues suggest that, while coverage of pregnant women and children should still be prioritized, wide-scale communal use of insecticide-treated bed nets would provide considerable benefit to vulnerable groups and should be promoted and evaluated in the field.

## Editors' Summary

**Background.:**

Malaria—a parasitic disease common in tropical and subtropical countries—causes about a million deaths every year, mainly among young children and pregnant women living in sub-Saharan Africa. The parasite responsible for most of these deaths is Plasmodium falciparum. Like all malaria parasites, it has a complex life cycle, part of which takes place inside mosquitoes. When a malaria-carrying mosquito bites a person (usually at night), parasites enter the human blood stream and travel to the liver where they reproduce before invading red blood cells. Here, they multiply again before bursting out and infecting more red blood cells as well as causing a high fever and sometimes damaging the nervous system, liver, and kidneys. When a mosquito bites someone who is infected, it often picks up parasites in its blood meal (mosquitoes need mammalian blood for successful reproduction), thus completing the malarial transmission cycle.

**Why Was This Study Done?:**

One way to break this cycle and reduce malarial transmission is to decrease the number of contacts between people and mosquitoes by encouraging people to sleep under insecticide-treated nets (ITNs). Field studies have shown that ITN use reduces deaths from malaria, so the Roll Back Malaria Partnership, the United Nations Millennium Development Goals, and the US President's Malaria Initiative have set a target of at least 80% use of ITNs by young children and pregnant women (the people most vulnerable to malaria) by 2010. But would broader population coverage with ITNs interrupt the malarial transmission cycle more effectively? Might the wider use of ITNs (which both directly kill mosquitoes and repel them so that mosquitoes have to travel farther to get the blood they need to reproduce) be a more effective way to reduce local mosquito numbers and, consequently, human–mosquito contacts and malarial transmission? In this study, the researchers used mathematical modeling to ask how much community-wide coverage with ITNs is needed to protect vulnerable individuals against malaria without them personally using an ITN.

**What Did the Researchers Do and Find?:**

The researchers developed a model for the transmission of the malaria parasite using data collected in Tanzanian villages where malaria is common, and used it to investigate how different patterns of ITN use might affect the individual protection of ITN users and the communal protection of nonusers. High (80%) coverage targeted at young children and pregnant women (less than 20% of the population) provided limited but valuable protection to these vulnerable individuals. However, the model predicted that a similar degree of community-wide protection would result if 35% of the whole population slept under ITNs provided there was no nonhuman blood supply for the mosquitoes. In other words, the vulnerable individuals in the population received the same degree of protection from this intervention as they would have got from personally using an ITN. If an alternative blood supply for the mosquitoes (for example, cattle) was included in the model, just over half of the population needed to sleep under ITNs to provide the same degree of community-wide protection as targeted ITN use.

**What Do These Findings Mean?:**

Although the use of ITNs by vulnerable groups should remain a priority, these findings suggest that the wide-scale ITN use by the entire population should also be promoted. The use of ITNs by about half the population, predict the researchers, could protect all the young children in that population, even those who did not sleep under a net. As with all mathematical models, the accuracy of this prediction depends on the assumptions and data incorporated into the model. So before recommending community-wide use of ITNs, the actual level of communal protection provided by increased ITN coverage must be measured by rigorously evaluating ongoing national programmes. If such surveillance data confirm this model's predictions, wide coverage with ITNs might do more for public health in Africa than previously thought, suggest the researchers, provided the financial and logistical challenges associated with achieving high ITN coverage in this poor region of the world can be solved.

**Additional Information.:**

Please access these Web sites via the online version of this summary at http://dx.doi.org/10.1371/journal.pmed.0040229.

- The US Centers for Disease Control and Prevention provide information on malaria and on insecticide-treated nets (in English and Spanish)
- The MedlinePlus encyclopedia contains a page on malaria (in English and Spanish)
- Information is available from the World Health Organization on malaria (in English, Spanish, French, Russian, Arabic, and Chinese) and from the Roll Back Malaria Partnership on the use of insecticide-treated nets
- Information about the US President's Malaria Initiative is available, including a fact sheet on the use of ITNs

## Introduction

The massive malaria burden in Africa merits particular attention as the world struggles to realize a better life for the poorest [1,2]. The *Anopheles* mosquitoes that act as vectors for human *Plasmodium* parasites must access sugar, blood, and aquatic oviposition sites to complete their life cycle and maintain parasite transmission. The availability of such ecological resources to mosquitoes has long been recognized as a crucial determinant of malaria transmission [3], but quantitative understanding of this process, as well as viable means to prevent it, remain poorly developed compared with other disease [4] and pest systems [5]. Recent theoretical work highlights the enormous influence of blood source and aquatic habitat availability in determining malaria transmission intensity, disease burden, and their responsiveness to various forms of control [6–12]. Here we apply field-parameterized kinetic models of mosquito host availability [11,13] to identify important shortcomings of current global targets for delivering insecticide treated nets (ITNs) [2,14,15], the most important vector control tool in Africa today. Not only does the model outline the limitations of existing strategies that emphasize targeting of vulnerable groups such as young children and pregnant women [16–18], it also indicates how complementary strategies to promote coverage of whole populations, including nonvulnerable adults and older children [19], will achieve greater and more equitable reduction of disease burden than otherwise would be possible.

Insecticide-treated nets (ITNs) represent a practical and effective means to prevent malaria in Africa [20], so scaling up coverage to at least 80% use by young children and pregnant women by 2010 is a consensus target of the Millennium Development Goals (MDGs), the Roll Back Malaria Partnership, and the US President's Malaria Initiative [2,14,15]. Targeting individual protection to these vulnerable groups [16–18] is a well-founded and explicitly accepted priority of all three initiatives, because these groups bear the highest risk of morbidity and mortality from malaria. However, this strategy largely ignores the potentially greater community-wide benefits of broader population coverage [19], and no explicit resources, targets, or strategies have been proposed to achieve these benefits.

ITNs can protect not only the individuals and households that use them, but also members of the surrounding community [19,21–26]. This is because they kill adult mosquitoes directly or force them to undertake longer, more hazardous foraging expeditions in search of vertebrate blood and aquatic habits [11]. *Plasmodium falciparum,* the malaria parasite responsible for the bulk of deaths in Africa, requires at least 8 d to develop from imbibed gametocytes into mature sporozoites within the salivary glands of the vector mosquito. This means that most malaria transmission is carried out by mosquitoes that are at least 10 d old and have taken several previous blood meals at intervals of 2–5 d [27,28]. By even modestly increasing mosquito mortality while they attempt to feed on humans, ITNs can greatly reduce the number of mosquitoes that survive repeated hazardous encounters with protected humans [11]. Also, the excito-repellent properties of ITNs can reduce the frequency with which mosquitoes successfully acquire blood, often diverting them to feed on other mammals that do not host the malaria parasite, resulting in greatly reduced prevalence of sporozoite infection [11]. This theoretical rationale is strongly supported by detailed observations from experimental hut studies [29–34] and from larger village-scale trials: ITNs have been clearly shown to reduce malaria risk among unprotected individuals by suppressing the density [35–37], survival [35–37], human blood indices [38,39], and feeding frequency [39] of malaria vector populations.

Large reductions of transmission are required to appreciably reduce malaria burden in most of Africa [17,40], particularly in the longer term as exposure and immunity re-equilibrate [41]. ITNs can address this challenging need through direct personal protection and area-wide suppression of the malaria transmission intensity that benefits even nonusers. It has been suggested that such communal benefits can make large impacts on disease burden only if appreciable levels of coverage are achieved in the human population as a whole [11,12,19], but precise coverage targets for achieving this remain to be determined. So how much coverage is enough to protect individuals who do not use an ITN?

## Methods

### Overview

Here we used recently developed kinetic models of mosquito behaviour and mortality [11,13] to answer this question by considering the impact of ITNs on human host availability and feeding hazards to mosquitoes, as well as the consequences of such changes for malaria transmission intensity. Protection was estimated in terms of protection against exposure to infectious mosquito bites, expressed as the relative change in the entomological inoculation rate (EIR). EIR is a proven epidemiological indicator of malaria transmission intensity and a key determinant of disease burden [17,40].

Two common but ecologically distinct African malaria transmission systems are considered. First, we modelled an Anopheles gambiae Giles or *An. arabienis* Patton (sibling species from the same species complex known as *An. gambiae* sensu lato) population with access to human blood only. Second, we considered *An. arabiensis* populations in the presence of abundant cattle, which can act as alternative blood sources. *An. gambiae* greatly prefers humans, but *An. arabiensis* will readily feed upon cattle [42,43], so populations of these species respond quite differently to increasing ITN coverage, with malaria transmission by the latter typically being lower to begin with but less sensitive to control with ITNs [11].

In both transmission systems we considered ITNs with properties typical of those evaluated in rigorous clinical trials [20] or those of emerging technologies with improved operational durability [44–47]. Note that coverage is expressed as the proportion of the total human population using an ITN each night, rather than in terms of ownership, because this value is the most direct indicator of both personal and communal protection.

**Figure 1:**
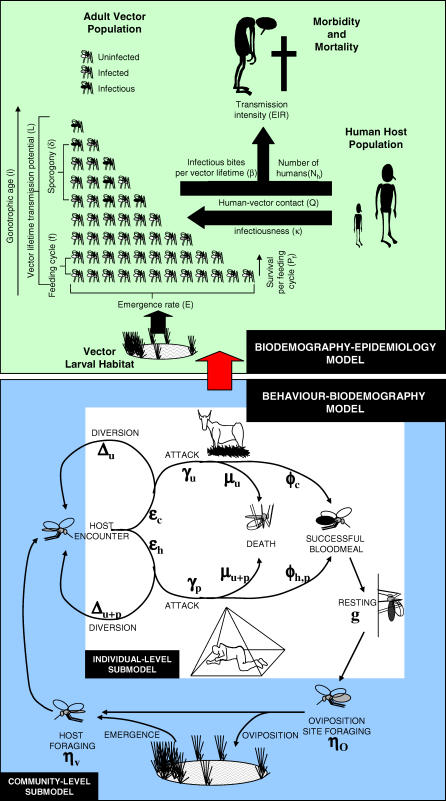
A Schematic Outline of the Two-Tier Model Used for This Analysis, Adapted from Previous Detailed Descriptions A detailed model of mosquito behaviour and survival as a function of host availability, ITN properties, compliance, and coverage [11,13] was used to estimate the key biodemographic parameters that determine malaria transmission intensity (behaviour–biodemography model). This model allowed the influence of ITN usage upon malaria transmission intensity to be estimated (biodemography–epidemiology model) in terms of EIR experienced by both users and nonusers [11,27,50]. All terms and symbols are defined in detail elsewhere [11,27,50,52] and are summarized in Methods.

Figure 1 provides an overview of how mosquito behaviour and survival were modelled as a function of host availability, ITN properties, compliance, and coverage. The approach described is essentially a behaviourally explicit extension of existing vector biodemography [48] models, which predict epidemiologically relevant outcomes such as exposure to transmission (the biodemography–epidemiology model). The principles and utility of the biodemography–epidemiology models we have used [27,49,50], as well as several others that are based on similar assumptions [6,18,28,51], are well established. Notably, this family of models realistically assumes that mosquito behaviour cycles between host seeking, feeding, resting, oviposition-site seeking, oviposition, and back to host seeking again [51]. Similarly to recent analyses of the importance of oviposition [7,8,10] and host acquisition [11,12] processes, here we explicitly modelled the underlying behavioural events that determine the input parameters of these biodemographic processes (the behaviour–biodemography model). Detailed consideration of mosquito behaviour and mortality upon encounter with individual hosts (the individual-level submodel) allows simulation of the impact of ITNs upon the foraging requirements and risks for mosquito populations at the community level (the community-level submodel). This hierarchical approach links individual- and community-level submodels into an integrated behaviour–biodemography model, which drives the outcome of the biodemography–epidemiology model and allows the influence of ITNs upon malaria transmission intensity to be estimated in terms of EIR experienced by both users and nonusers [11,27,50].

The specific modelling approach described here is almost identical to our recent exploration of the optimal properties of ITNs as a function of local ecology [11], apart from subtle improvements in terms calculating mosquito diversion, mortality, and feeding probabilities per host encounter. It is also similar to and consistent with the approaches of others [6,12] but accounts for the fact that ITNs can act only during times of the night when they are actually in use, so that their overall protection is also influenced by subtle variations in the behavioural interactions between humans and mosquitoes [13]. This model has already been evaluated through improved iterations in terms of sensitivity to variations in the assumed parameter values for the insecticidal and excito-repellent properties of ITNs [11], the survival rate of mosquitoes while foraging for resources [11], the innate resource preferences of vector populations [11,50,52], and the availability of those resources, including oviposition sites [50] and alternative blood meal hosts [11,50].

While the analysis outlined here could be implemented with either of the recently developed (and perhaps more elegant) alternative models [6,12], this particular form captures all of the same processes without necessitating the mathematical subtleties of integration, differentiation, equilibrium analysis, or limits. While these are inherently valuable tools for mathematical modelling, they often constitute “black boxes” to nonmathematicians, including several authors of this article. We therefore chose a model that does not require mathematical complexities that might limit accessibility to some of the field biologists and epidemiologists for whom this analysis is most relevant. The model is presented as a downloadable spreadsheet (see Protocol S1) and has proven valuable for teaching the ecological basis of malaria epidemiology and control to students in both the developed and developing world.

### Modelling Mosquito Behaviour and Mortality at the Individual Level

Here we describe a submodel of behavioural and mortality processes that occur at the level of individual mosquitoes seeking, encountering, attacking, and feeding upon individual blood hosts. Another important simplification to consider is that, like most deterministic malaria transmission models, our approach assumed a “malaria in a bottle” scenario in which populations of identical parasites, vectors, and hosts are mixed homogenously within an enclosed system [53]. One important corollary of this assumption is that well-established variations of vulnerability to malaria infection within human populations [16,17] or associated variations in attractiveness and availability to mosquitoes [9,54–56] are not explicitly modelled.

As defined previously [52], the availability *(a)* of any host *(j)* of any species *(s)* is the product of the rate at which individual vectors encounter it *(ɛ_s,j_)* and the probability that, once encountered, they will feed upon it *(φ_s,j_):*

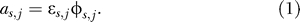



Note that this kinetic definition of *availability* as a rate per unit time is consistent with applications of the same term to acquisition of oviposition sites [10], the term *attraction rate* for blood sources [6,57], and the terms *feeding rate* and *oviposition rate* for both resources [8,12].

We considered successful feeding as just one of three possible outcomes of a host encounter by a female vector, the other two being death while attempting to feed and diversion to seek another host (Figure 1). We considered this a two-stage process in which the vector first either attacks the encountered host or is diverted away and searches for another, the probabilities of which we denote as *γ* and *Δ,* respectively. This definition of diversion includes the combined effects of noncontact repellency and contact-mediated irritancy, often referred to as excito-repellency [58,59]. Considering mean values for hosts of any given species *(s),* the sum of these two probabilities is:

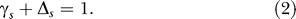



We then considered the second stage of the blood acquisition process, namely feeding. Knowing the probabilities that the vector will either feed successfully *(φ_s_)* or die in the attempt *(μ_s_)* per attack (rather than per encounter) allowed us to calculate the probability of a successful feed per encounter:

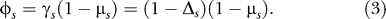



Specifically, the cases of cattle *(c)* and unprotected humans *(h,u)* were dealt with in a straightforward manner as follows, where *Δ_u_* and *μ_u_* represent a common parameter value for both types of host (Table 1):

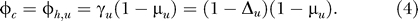



**Table 1:**
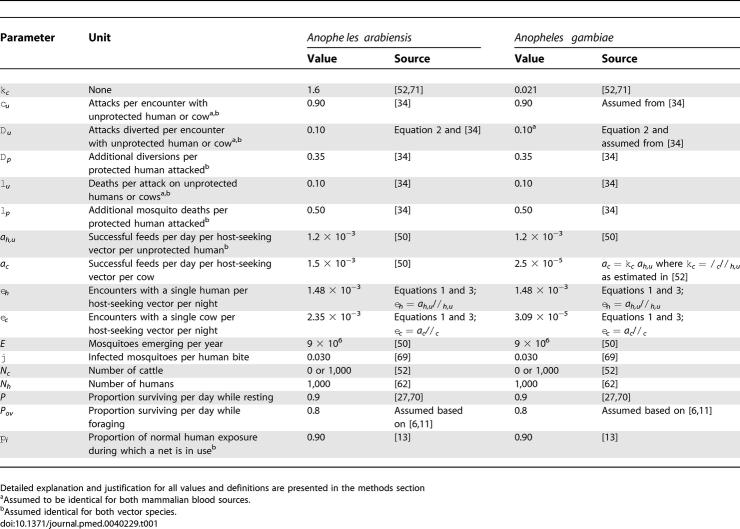
Behavioural and Host Availability Input Parameters for Both Vector Species

Personal protection measures such as bed nets, repellents, or domestic insecticide use were envisaged as three possible outcomes, the probabilities of which sum to 1: For a vector that would normally choose to feed upon an encountered unprotected human with a probability of *φ_h,u_,* the presence of a net or other intervention is expected to influence this probability for protected humans *(φ_h,p_)* as a function of the excess probability of diverting *(Δ_p_)* and killing *(μ_p_)* that vector (Figure 1). The combined baseline and net-induced probabilities of diversion *(Δ_u_*_ + *p*_ *)* or mortality *(μ_u + p_)* were calculated as follows:

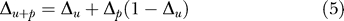
and

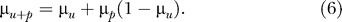



These parameters allowed us to calculate the feeding probability for a human who always uses and is protected by a net *(φ_h,p_):*

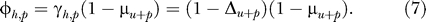



These equations are parameterized using data from experimental hut trials in which the human participants slept within the net throughout the period of data collection (Table 1). However, very few human beings spend their entire day asleep or using a net [13] so the true probability of feeding upon a typical net user (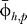 ) is calculated by weighting *φ_h,u_* and *φ_h,p_* according to the proportion of normal exposure during which the host is actually covered *(π_i_):*

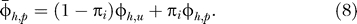



Equations 5-7 differ slightly from those previously proposed [11], which treated diversion and killing as independent events, conditional on the host having and using a net. At low values of *π_i_* these changes relative to [11] make little difference, but the model described here is more realistic at high values of *π_i_*.

### Extrapolating Impacts of Insecticide-Treated Nets to the Community Level

Given the above submodel for the interactions of mosquitoes with individual mammalian hosts, it was possible to extrapolate the likely large-area effects of these small-scale influences on entire vector populations and the human communities they feed upon.

For any given number of cattle *(N_c_),* unprotected humans *(N_h,u_),* and protected humans *(N_h,p_),* the mean seeking interval for vertebrate hosts *(η_v_)* can be calculated as the reciprocal of total host availability *(A)* [52], using estimates of these feeding probabilities and their corresponding encounter rates, adapting Equation 1 from our original formulation [50]:

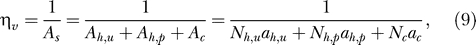
where *A_s_* refers to the total availability of all hosts of species *s.* In this case, the species or species categories considered were unprotected humans *(h,u),* protected humans *(h,p),* and cattle *(c).* Values for *a_c_* and *a_h,u_* (previously *a_h_* [50]) were estimated exactly as described previously [50] and *a_h,p_* was calculated as follows:

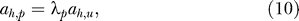
where *λ_p_* is the relative availability of protected versus unprotected hosts, estimated in terms of the ratio of their feeding probabilities:

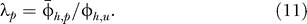



Foraging for resources is an intrinsically dangerous undertaking for mosquitoes, and it is commonly assumed that survival during these phases is lower than while resting in houses [6,60]. We adapted Equation 3 from our previous formulation [50] to estimate the survival rate per feeding cycle *(P_f_)* as the product of the probability of surviving the eventual attack on a host that may be protected *(P_γ_)* and the probabilities of surviving the gestation *(g),* oviposition site-seeking *(η_o_),* and vertebrate host-seeking *(η_v_)* intervals, with distinct daily survival probabilities for the resting *(P),* foraging for either oviposition sites or vertebrate hosts *(P_ov_),* and attacking *(P_γ_)* phases:

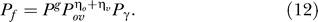



The mean probability of mosquitoes surviving their eventual chosen host attack *(P_γ_)* was calculated assuming that the proportion of all attacks that end in death is the sum of the mortality probabilities for attacking protected and unprotected hosts, weighted according to the proportion of all encounters that will occur on such hosts. Assuming that protection does not affect encounter rates, and that these rates are proportional to availability when unprotected, we applied this weighting approach to estimate total attack-related mortality rate and consequent survival as follows:

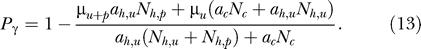



Similarly, the human blood index is calculated as the proportion of total host availability accounted for by humans [52], similarly to Equation 9:

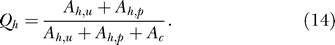



The EIR for protected and unprotected individuals was then calculated from the total number of infectious bites upon humans that occur in the population as a whole *(β E)* [27,49], the share of the total human availability represented by that group, and the population size of that group:

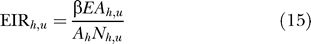


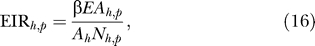
where *β* is the mean number of infectious human bites each emerging mosquito takes in its lifetime and *E* is the emergence rate of mosquitoes [27]. Dividing Equation 16 by Equation 15, substituting with Equation 10, and rearranging also leads to an intuitively satisfactory solution, consistent with independently formulated models of personal protection [13]:

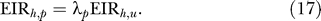



Otherwise, we modelled malaria transmission exactly as previously described [50]. Note that this model has been adapted [11,50] from its original formulation [27] to account for superinfection of mosquitoes [28] and daily time increments to smooth the effects of changing host availability patterns on feeding cycle length [50]. For ease of comparison and interpretation, the impact of ITNs is presented in terms of the relative transmission intensity EIR*_C_*/EIR*_0_* at a given coverage level (*C;* note distinction from *c,* which denotes cattle hosts) as a result of personal and communal protection amongst users and nonusers:

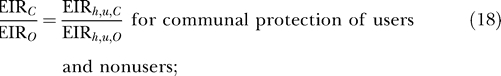


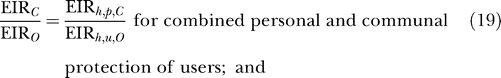


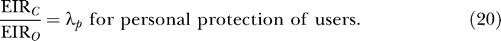



### Baseline Mosquito Behaviour, Host Availability, and Survival Parameters

The parameter definitions and values used to implement this analysis are summarized in Table 1. Namwawala, in the Kilombero Valley, southern Tanzania is the primary centre for parameterising our model because of the exceptionally detailed quantitative characterisation of malaria transmission and vector biodemography in this village and the surrounding area. This is a holoendemic village with intense seasonal transmission, stable high parasite prevalence in humans, and a heavy burden of clinical malaria [61–68]. At this site the bulk of transmission is mediated by *An. gambiae* sensu lato (of which the main species involved in transmission is *An. arabiensis*) and transmission intensity has been modelled with available field data [27,49].

As previously described [27,49], we based our estimate of human population size [62] approximately upon those reported for this particular village during the early 1990s. Nevertheless, we used a human population size of 1,000 and, where relevant, a bovine population of the same size so that the EIR experienced by users and nonusers could be easily calculated at net coverage levels approaching 0% and 100%. By setting coverage to 0.001 or 0.999, this model simulates a single user or nonuser in the population, respectively.

Infectiousness of humans *(κ)* is set to 0.030, reflecting a more precise recent estimate [69] than was available previously [61,63]. In a typical holoendemic scenario, the infectiousness of the human population is thought to be largely insensitive to reductions in transmission intensity [69]. In the interests of making conservative and generalizable predictions, we assumed that increasing coverage with ITNs will not affect *κ* [69], even though reduction of *κ* is likely at EIR values below 10 infectious bites per person per year [56].

We set mean daily survival of the resting phase *(P)* at 0.90, reflecting a median value of daily survival at four well-characterised holoendemic sites [27] and estimated daily indoor survival for *An. gambiae* s.l*.* in Tanzania [70]. As previously described, the daily survival rate of mosquitoes while foraging for blood or oviposition sites *(P_ov_)* was set at 0.80, representing a median value of plausible field values [11]. The results of experimental hut studies [34] were combined with host-choice evaluations [71] and appropriate analytical models [50,52] to define the attack and mortality probabilities of *An. arabiensis* encountering cattle or humans: we set the probability that *An. arabiensis* will attack unprotected cattle or humans *(γ_u_),* conditional upon encountering them, to be 0.90 and the chance that they will die in the attempt *(μ_u_)* at 0.10.

Using these parameters and Equation 3, we calculated that, for *An. arabiensis,* the overall feeding probability upon either cattle *(φ_c_)* or unprotected humans *(φ_h,u_)* would be 0.81, a value similar to previous estimates of approximately 0.80–0.85 for the feeding success of *An. gambiae* sensu lato on sleeping humans in Tanzania [34,62]. We also applied these same probabilities of attacking *(γ_u_),* feeding *(φ_h,u_),* and dying *(μ_u_)* to *An. gambiae* sensu stricto encountering unprotected humans. The availabilities of unprotected humans and cattle were calculated for *An. arabiensis* using field measurements of the duration of the feeding cycle and were extended to *An. gambiae* s.s., accounting for the lower estimated relative availability of cattle *(λ_c_)* to this mosquito species as previously described [52]. Note that *λ_c_* is assumed to modify *a*_c_ by affecting the encounter rate only, indicating that these mosquitoes can differentiate between preferred and nonpreferred hosts at long ranges [72–74]. In the case of *An. arabiensis* this assumption is consistent with the longer spatial range of attraction of cows relative to humans for zoophilic members of the *An. gambiae* complex [72–74].

### Parameters Reflecting the Effects of Insecticide-Treated Bed Nets

**Figure 2:**
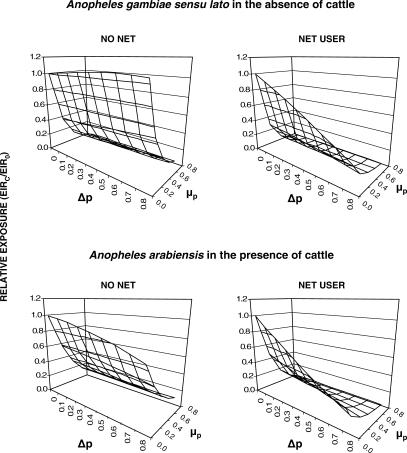
The Simulated Protection ITNs Afford against Exposure to Malaria Transmission as a Function of Their Ability to Divert and Kill Host-Seeking Mosquitoes Protection is expressed as relative exposure to malaria transmission (EIR*_C_*/EIR*_o_*) for individuals with (Equation 19) and without (Equation 18) nets is plotted as a function of their ability to divert *(Δ_p_)* and kill *(μ_p_)* mosquitoes attacking protected humans. To simulate the likely field properties of existing long-lasting insecticidal nets with a full range of insecticidal and excito-repellent properties, the parameters of this model reflecting increased mosquito mortality *(μ_p_)* and diversion *(Δ_p_)* were varied across a plausible range of 0–0.8. As described in the main text and previous publications, these results represent simulations in two distinctive scenarios: *An. gambiae* sensu lato in the absence of cattle (results for both sibling species are identical) and *An. arabiensis* in the presence of one head of cattle per person. The biodemographic parameters of the interacting vector and parasite are also exactly as described previously [11,13] with survival of foraging mosquitoes *(P_ov_)* set at 0.8 per day. Coverage levels of 75% net usage was assumed, consistent with the results of surveys in the Kilombero Valley, southern Tanzania (see Methods: Parameters Reflecting the Effects of Insecticide-Treated Bed Nets).

The parameter definitions and values describing the impacts of ITNs on vector behaviour and mortality at the level of individual interactions are listed in Table 1. The impacts of ITNs very much depend on their excito-repellent and insecticidal properties, which are most representatively evaluated using well-established experimental hut methodologies [59,75,76] that have been extensively applied to this particular intervention [29–34]. Furthermore, the interaction of these two properties, to yield varying levels of personal and communal protection, is complex and has crucial implications for ITN programmes across Africa [11]. Sensitivity analysis of models similar to those used in this paper [11] have previously been used to explore the influence that these properties might have upon the magnitude and equity of protection afforded by ITNs (Figure 2). In order to validate this slightly revised model (see Equations 4-8) and similarly investigate such interactions at ITN coverage levels that can be plausibly sustained, we examined usage data collected during routine socioeconomic status surveys of a long-standing demographic surveillance system in the Kilombero Valley, southern Tanzania, where social marketing programmes have been well established since 1997 [77,78]. Data from the annual ITN usage survey in 2004 were used because they overlap with detailed entomological surveys of malaria transmission (which will be reported elsewhere). These surveys of randomly sampled residents from across two rural districts indicate that 75% (11,982/16,086) net use was achieved although most of these nets were not effectively treated [79]. In this sensitivity analysis, we assumed that new long-lasting ITN technologies [44–47] will enable sustained coverage with nets that are effectively treated even under the most rigorous programmatic field conditions.

Figure 2 shows that, for the comparatively zoophilic vector *An. arabiensis,* in the presence of alternative hosts, excito-repellency consistently enhances the benefits for both users and nonusers, regardless of the insecticidal properties of the net. Consistent with previous analyses using this model [11], this simulation suggests that nets that are purely excito-repellent and lack insecticidal properties could slightly increase exposure of nonusers to *An. gambiae* sensu lato by diverting mosquitoes to them where no alternative sources of blood are available. Thus, purely diversionary vector control strategies may indeed be ethically questionable, as was previously suggested [31,34,80,81]. Nevertheless, even modest insecticidal properties are expected to counterbalance this inequity and confer a useful communal reduction of EIR. While repellent properties do slightly reduce the benefits to nonusers exposed to anthropophagic vectors lacking an alternative host, this slight disadvantage is likely to be outweighed in practice by the advantage of improved personal protection for users: Excito-repellent properties and physical barriers add to the effectiveness of insecticides for personal protection because these two incentives constitute the major motivating force behind ITN uptake and use at the individual and subsequently the community level. It is also reassuring to note that the predictions and epidemiological implications of this slightly revised model are very similar to those reported for its previous iteration [11].

We therefore concluded that the simulations described in the main text should consider ITNs with both insecticidal and excito-repellent properties, consistent with those of products currently on the market that have been evaluated in a variety of settings and experimental designs.

To simulate the likely properties of established ITNs under programmatic conditions, we conservatively assumed they will both divert and kill 40% more mosquitoes than an unprotected human (*μ_p_* = 0.4 and *Δ_p_* = 0.4). A net with such proper-ties would protect against 64% of indoor exposure (1 − [(1 − 0.4) × (1 − 0.4)] = 0.64), as measured in a typical experimental hut trial [46,76]. To explore the best possible future scenario for the development of highly durable ITNs [44–47] or regular retreatment services [82], we also simulated increasing co-verage with nets that divert and kill 80% more mosquitoes than with an unprotected human (*μ_p_* = 0.8 and *Δ_p_* = 0.8), providing 96% protection (1 − [(1 − 0.8) × (1 − 0.8)] = 0.96). The proportion of normal biting exposure that occurs while nets are actually in use *(π_i_)* has been estimated as 90% for A. gambiae in southern Tanzania [13], so we set *π_i_* to a value of 0.90.

## Results

**Figure 3:**
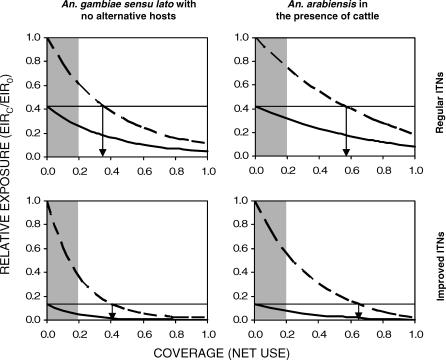
Relative Exposure to Malaria Transmission (EIR*_C_*/EIR*_o_*) as a Function of Increasing Coverage with Insecticide-Treated Nets We express coverage as the proportion of the total human population using an ITN each night, and protection as the proportional reduction of infectious bites to which a resident is exposed (see Methods). Individual protection afforded to users (thin solid line; Equation 20) and communal protection afforded to nonusers (thick dashed line; Equation 18), as well as their combined effect on users (thick solid line; Equation 19) are separately calculated [11,13]. Two distinct but common and broadly distributed ecological scenarios in Africa are considered: (1) *An. gambiae* or *An. arabienis* (sibling species of the same species complex known as *An. gambiae* sensu lato) populations in the absence of alternative blood sources and (2) vector populations dominated by *An. arabiensis* in the presence of abundant cattle as alternative hosts. Both scenarios are simulated with ITNs that have either standard or improved properties (See Methods). Grey shading represents an approximate absolute maximum for community-level coverage achievable by covering vulnerable under five years of age and pregnant population groups only with perfect targeting efficiency. Arrows extrapolate the thresholds at which communal and personal protection are equivalent.

Figure 3 illustrates how increasing community-level protection of ITN nonusers and users alike combines with constant individual protection to reduce exposure to malaria. Regardless of vector species or the availability of alternative hosts, modestly effective conventional ITNs achieve much greater impact upon human exposure, even that of users, if approximately half or more of the whole human population is covered. While this principle has already been suggested by field trials [19] and two independently formulated models [11,12], here we have identified specific coverage thresholds at which communal protection becomes greater than or equal to individual personal protection. Where alternative hosts for vector mosquitoes are absent, 35% of the human population must sleep under regular ITNs to achieve equivalence of personal and communal protection mechanisms, resulting in major community-wide suppression of exposure. The same target is achieved at 55% coverage where alternative hosts such as cattle are present.

The insecticidal and excito-repellent properties of ITNs that define levels of personal protection also determine the extent of community-wide alleviation of exposure amongst users and nonusers alike [11], so improved ITN properties consistently result in improved overall impact. In our model, slightly higher usage rates were required to achieve equivalence of individual and communal effects, with thresholds of 40% and 64% coverage for vector populations with and without alternative hosts, respectively (Figure 3). While emerging ITN technologies with long-lasting insecticidal properties under programmatic conditions [44] would confer useful personal protection even at low coverage levels, personal protection was greatly enhanced by communal protection. At the 75% total population coverage recently achieved with largely untreated nets in southern Tanzania (Killeen et al., unpublished data), net users and nonusers are predicted to receive >98% and >90% protection, respectively, regardless of ecological scenario, if those nets were to be replaced with improved long-lasting insecticidal nets. Even for users of improved ITNs, this level of protection against African vector species is impossible without the contribution of community-level transmission suppression, because at least 10% of exposure occurs outdoors during times of the night when nets are not in use [13,83]. We conclude that modest coverage (thresholds of approximately 35%–65% use, depending on ecological scenario) of entire malaria-endemic populations, rather than just the most vulnerable minority, is needed to realize the full potential of ITNs, even with longer-lasting products or regular retreatment services [14,44]. This range of modelled thresholds is remarkably consistent with the figure of 50% suggested by large-scale field trials using approximately equivalent technology [19].

## Discussion

In addition to the direct impacts on vector populations explicitly modelled above, coverage of adults and older children is likely to have further benefits arising from subtleties of mosquito resource utilization that are often under-appreciated. Over 80% of human-to-mosquito transmission originates from adults and children over five years of age, because these groups constitute the bulk of the population and are more attractive to mosquitoes [56]. Where the entomological inoculation rate is fewer than ten infectious bites per person per year, the distributions of infectiousness [56,69], morbidity, and mortality will all shift into these older age groups, necessitating protection of all members of the population. Under such conditions, ITNs could suppress transmission not only through direct impacts on mosquito mortality, host choice, and feeding frequency [11], but also by limiting the prevalence, density, and infectiousness of malaria parasites in the human population [56].

An under-emphasized feature of communal protection is the enhancement of ITN programme equity, regardless of ecological scenario or ITN effectiveness: If the majority of people living in malaria-endemic Africa regularly used existing ITN technologies, nonusers would receive communal protection at least equivalent to using the only ITN in an otherwise unprotected population (Figure 3). This means that all children would equitably receive communal protection at least equivalent to the personal protection of an ITN, with users receiving multiplicative combined effects on exposure of both personal and communal benefits. While the wisdom of targeting interventions to protect at-risk individuals is based on solid scientific grounds [9,18,84] and is widely accepted [16], this approach should not preclude efforts to maximize communal protection through less selective delivery mechanisms. Targeting limited subsidies to maximize personal protection of the most vulnerable should remain a priority, but more equitable and effective suppression of risk for entire populations, including vulnerable groups, can be attained with quite modest coverage across all ages. Most field evaluations of ITNs have been conducted at reasonably high coverage levels [19], and all five mortality trials [21,85–88] that estimated that ITNs save 5.5 lives for every 1,000 children protected [20] covered large portions of entire communities rather than only the children themselves.

The choice of ITN delivery strategy has proven contentious in recent years [89,90], but proponents of both market-based and public-sector approaches equally emphasize targeting strategies [9,16,84] to enhance equity and minimize leakage of subsidized ITNs beyond intended target groups [91–94]. While optimal targeting of finite subsidies is highly desirable, there are fundamental limitations to the impact that can be achieved: Even if resources were perfectly targeted, 80% coverage of pregnant women and children under five years of age could be accomplished with less than 20% coverage of the whole population, and even less of the total human host availability [11,56], as well as the infectious parasite reservoir [56,69]. Even if the ITN coverage targets of the MDGs were attained with flawless targeting efficiency, the substantial and equitable benefits of communal protection would not be achieved. Specifically, the target of 70% less exposure to transmission [13] would not be attained by the remaining minority of vulnerable individuals who are not covered and do not use an ITN, regardless of ecological scenario or ITN properties (Figure 3). We therefore highlight an important caveat to the following conclusion of the current Global Strategic Framework for ITN scaleup in Africa [95]: “In order to achieve maximum public health impact, ITN coverage needs to be maximized amongst those population groups that are most vulnerable to malaria infection and its consequences, primarily pregnant women and children under five years of age.”

Specifically, we conclude that protecting the vulnerable can achieve maximum public health impact only if complemented by strategies that also achieve broad coverage of the population as a whole.

In reality, the targets for coverage of vulnerable groups will not be reached without some leakage and inequity. Our analysis suggests that such concerns may be less of a problem than the targets themselves and may be minimized by extending coverage priorities to include all age groups. Fortunately, consensus is finally emerging that a range of approaches to ITN deployment merit investigation, development, and comparative evaluation at scales for which no precedent yet exists [95]. Note that this analysis supports the implementation of any of the diverse and rapidly emerging delivery strategies as long as high coverage with long-lasting ITNs is sustained across entire malaria-endemic populations on national scales. Perhaps the most important remaining question is: How can such population-wide coverage levels be affordably and cost-effectively sustained?

Growing financial support for malaria control globally [14,15,95] may enable fully subsidized provision to entire populations [82] of the world's most impoverished, malaria-afflicted nations. Existing evidence, based largely on individual protection alone, indicates that ITNs are as cost-effective as childhood immunization [96], and future analyses should explicitly consider the additional benefits of communal protection. Implementing this goal may be relatively straightforward for programmes that are primarily subsidized and implemented through the public sector, such as recent successful initiatives associated with vaccination campaigns [91]. By comparison, social marketing approaches, including hybrid systems that deliver public subsidies through the private sector, may require more detailed consideration, particularly where cost sharing with the target population is substantial and biased toward the nonpregnant adults and older children who are key to communal protection.

Although social marketing approaches to ITN distribution face substantial challenges [93,97,98], notable success in terms of coverage and impact have been reported in a variety of settings [94,99,100], including the KINET programme in Kilombero Valley, southern Tanzania where ITNs have been promoted and subsidized since 1996 [77,78]. Much of the essential experience generated by KINET was later integrated into the ITN promotion strategy of the National Malaria Control Programme of Tanzania, which supports private sector distribution through a voucher system that subsidizes purchase by vulnerable priority groups [101]. In the meantime, the preceding KINET pilot in Kilombero has achieved 75 % net use amongst randomly sampled residents of all ages (Killeen et al., unpublished data). It is particularly noteworthy that substantial levels of communal protection were achieved [102] (unpublished data) even though most of these nets were untreated or poorly treated at the time of evaluation [79] (unpublished data). Reassuringly, the model applied here approximately reproduces these patterns of communal protection using plausible parameter estimates for the net properties, vector behaviours, and host demographics of the area (unpublished data). We therefore recommend that the cost-effectiveness of such hybrid approaches be explicitly evaluated in terms of the complementary respective contributions of public-sector subsidies and cost-sharing by target populations to personal and communal protection.

While appropriate engagement and sensitization of malaria-afflicted populations is essential to the success of any ITN promotion programme, this is likely to be especially true where cost-sharing by the target population will be needed to complement limited public subsidies. Such cost-sharing schemes may be the only affordable means to support full population coverage where available subsidies are inadequate. In such resource-limited circumstances, high levels of awareness, acceptance, and willingness to pay will be essential to enable concerted use of ITNs by adults and shared protection of all children within their communities.

Overly confident extrapolation from mathematical models to set operational targets for malaria control has proved to be a grave mistake in the past [103]. A number of complications not captured by this model could emerge as ITN coverage increases, not least of which might be increased selection for insecticide resistance [104,105]. While we urge caution in interpreting the numerical results of our analysis, the phenomenon outlined is well established and has clear implications for malaria control in Africa and beyond [19]. In fact, the analysis presented here provides a generalizable rationale that strongly supports the conclusions of the most recent and meticulous evaluations of the community-level benefits of ITNs: “High coverage with ITNs will do more for public health in Africa than previously imagined” [19].

We therefore suggest that further field data, analyzed with appropriate theoretical models and cost-effectiveness frameworks, are required to verify and quantify the levels of communal protection afforded by increasing ITN use across Africa. International targets [2,14,15] should be amended to include thresholds for coverage of entire populations and monitored accordingly. By making life increasingly difficult for mosquitoes through programmes that promote ITN use by the majority of their human victims, it may be possible to protect the 15%–20% of children and pregnant women in African communities who would not otherwise be covered even if existing personal protection targets of the MDGs [2], the Roll Back Malaria Partnership [14], or the U.S. President's Malaria Initiative [15] were to be achieved.

## Supporting Information

Protocol S1 Model Spreadsheet A Microsoft Excel spreadsheet version of all model simulations presented here is available to download.
(1.1 MB XLS)

## Abbreviations

EIR: entomological inoculation rate
ITN: insecticide-treated net
MDG: Millennium Development Goals
